# Earliest Mexican Turkeys (*Meleagris gallopavo*) in the Maya Region: Implications for Pre-Hispanic Animal Trade and the Timing of Turkey Domestication

**DOI:** 10.1371/journal.pone.0042630

**Published:** 2012-08-08

**Authors:** Erin Kennedy Thornton, Kitty F. Emery, David W. Steadman, Camilla Speller, Ray Matheny, Dongya Yang

**Affiliations:** 1 Trent University Archaeological Research Centre, Trent University, Peterborough, Ontario, Canada; 2 Florida Museum of Natural History, University of Florida, Gainesville, Florida, United States of America; 3 Ancient DNA Laboratory, Department of Archaeology, Simon Fraser University, Burnaby, British Columbia, Canada; 4 Department of Anthropology, Brigham Young University, Provo, Utah, United States of America; 5 Department of Archaeology, University of Calgary, Calgary, Alberta, Canada; University of Florence, Italy

## Abstract

Late Preclassic (300 BC–AD 100) turkey remains identified at the archaeological site of El Mirador (Petén, Guatemala) represent the earliest evidence of the Mexican turkey (*Meleagris gallopavo*) in the ancient Maya world. Archaeological, zooarchaeological, and ancient DNA evidence combine to confirm the identification and context. The natural pre-Hispanic range of the Mexican turkey does not extend south of central Mexico, making the species non-local to the Maya area where another species, the ocellated turkey (*Meleagris ocellata*), is indigenous. Prior to this discovery, the earliest evidence of *M. gallopavo* in the Maya area dated to approximately one thousand years later. The El Mirador specimens therefore represent previously unrecorded Preclassic exchange of animals from northern Mesoamerica to the Maya cultural region. As the earliest evidence of *M. gallopavo* found outside its natural geographic range, the El Mirador turkeys also represent the earliest indirect evidence for Mesoamerican turkey rearing or domestication. The presence of male, female and sub-adult turkeys, and reduced flight morphology further suggests that the El Mirador turkeys were raised in captivity. This supports an argument for the origins of turkey husbandry or at least captive rearing in the Preclassic.

## Introduction

The turkey was a significant animal for the ancient Maya, whose realm stretched from northern Honduras to southern Mexico. Turkeys were not only a source of food, but were also important sacrificial offerings, and their feathers, bones, and other byproducts were used to produce medicines, fans, tools, musical instruments and personal adornments. Until this study, however, the Maya were assumed to have used only the native, wild ocellated turkey (*Meleagris ocellata*) throughout the Preclassic to Classic period of cultural florescence (ending in AD 1000). The Mexican turkey (*Meleagris gallopavo gallopavo*), domesticated in central/northern Mexico [1], was presumed to have been introduced fairly late in time during the Postclassic (AD 1000–1500), the final period of pre-Contact Maya occupation (Table S1). Our recent identification of *M. gallopavo* in Late Preclassic (*ca.* 300 BC–AD 100) deposits from the Maya archaeological site of El Mirador overturns these assumptions and places *M. gallopavo* introduction 1000 years earlier. In this collaborative study, we identified the El Mirador turkey specimens through morphology, osteometrics, and ancient DNA (aDNA) analysis. The context and dates were confirmed through archaeology and AMS radiocarbon dating. The results lead us to reconsider the timing of turkey domestication and diffusion throughout Mesoamerica, as well as the nature and extent of Preclassic Mesoamerican trade connections.

**Figure 1:**
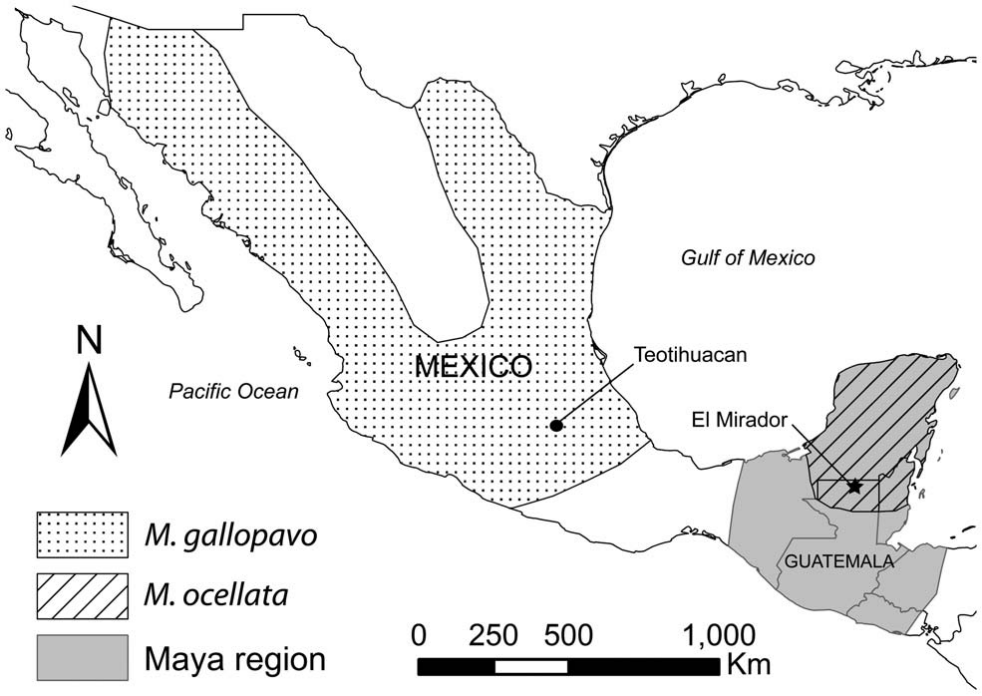
Pre-Hispanic range of *M. ocellata* and *M. gallopavo* [**2]**, [6] in Mesoamerica, and location of discussed archaeological sites.

Today, the domesticated form of *M. gallopavo* is distributed worldwide, but its wild progenitor was limited to the eastern and southwestern United States and central/northern Mexico north of the Isthmus of Tehuantepec, and thus outside the Maya cultural region [1]–[3] (Fig. 1). The absence of wild populations of *M. gallopavo* in the Maya area after the end of the Pleistocene is supported by both the paleontological and archaeological records [4]–[6]. In contrast, the ocellated turkey ranges throughout the northern half of the Maya cultural area including Mexico's Yucatan Peninsula and northern Belize and Guatemala where it remains locally common [3], [7]. Although some ocellated turkeys may have been raised in captivity during pre-Hispanic times, there is no evidence that this species was ever domesticated [2], [8].

The exact timing and location of New World turkey domestication are still unknown: recent evidence points to at least two separate domestication events in northern or central Mexico and the North American Southwest [9]. In central Mexico, archaeological *M. gallopavo* bones have been identified at sites dating to 800–100 BC [10], [11]. It is unclear whether these early specimens represent wild or domestic individuals, but domestic turkeys were likely established in central Mexico by the first half of the Classic Period (*ca.* AD 200–1000) [12]. Until this study, *M. gallopavo* had not been identified in any Maya archaeological deposits predating the Postclassic [6], [8], [13]. The Postclassic Maya specimens are all presumed to represent domesticated individuals [8], [13] either imported directly from central/northern Mexico or bred and raised in the Maya world following their initial introduction.

The *M. gallopavo* specimens reported here were recovered from the major archaeological site of El Mirador, located in north-central Petén, Guatemala (Fig. 1, Text S1). Settlement at the site dates back to at least 600 BC, but population and architectural extent peaked at the site during the Late Preclassic (300 BC–AD 100) when one of the largest assemblages of Maya public architecture was constructed at the site, including but not limited to the Tigre and Danta Pyramids. El Mirador's Late Preclassic florescence coincided with a time of increasing social, political, and economic complexity in the Maya region when many of the hallmarks of Classic Maya civilization (e.g., institution of kingship, monumental stone architecture, extensive trade networks, and elaborate iconography) were established. At the end of the Late Preclassic, the site was largely abandoned. Although there was a small presence in the Early Classic and somewhat more substantial settlement during the Late Classic, no monumental constructions like those from the Late Preclassic occurred during these later occupations.

**Figure 2:**
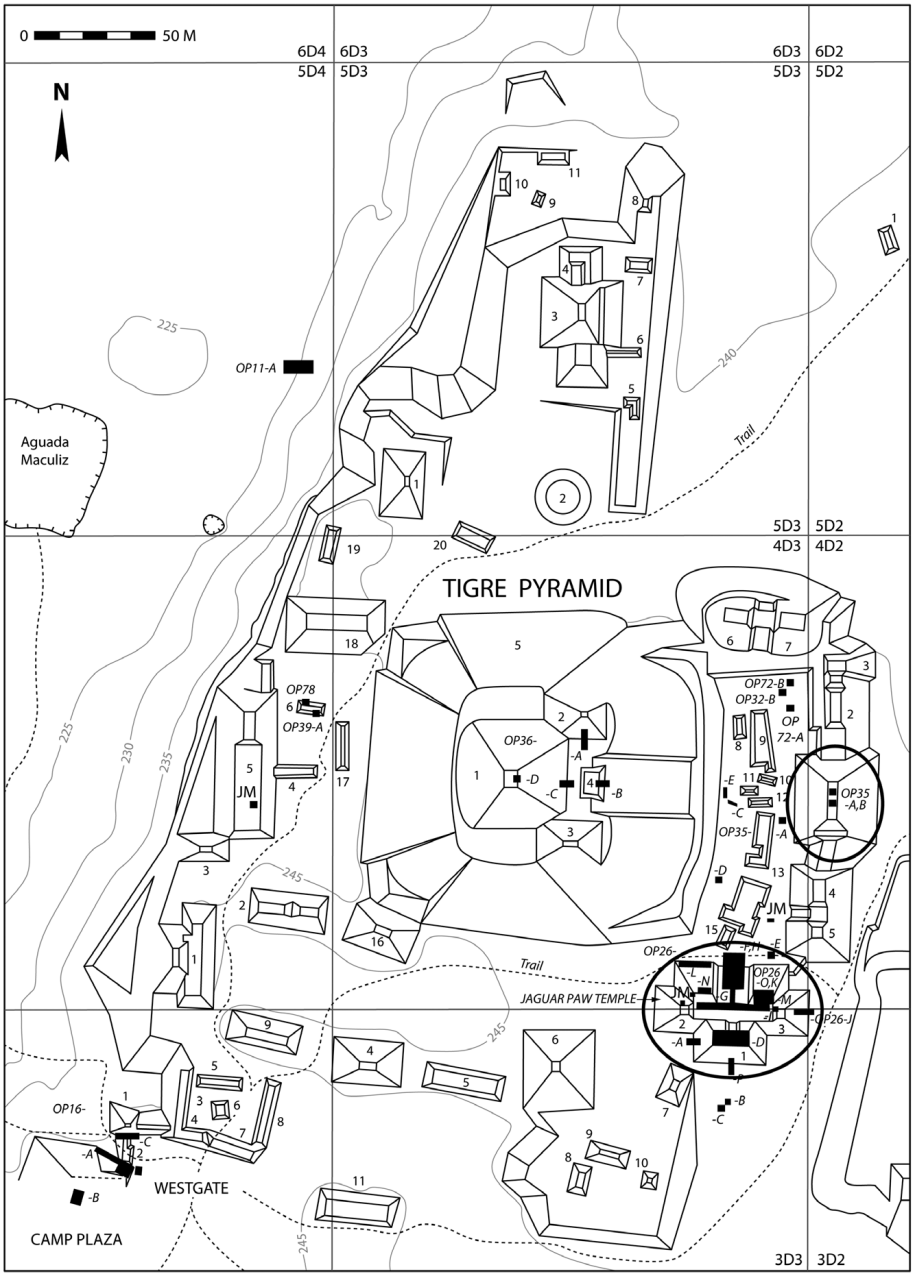
El Tigre Complex showing structures containing turkey bones (circled). Redrawn from original by B. Dahlin.

Zooarchaeological turkey specimens (n = 7) from El Mirador were recovered along with other animal remains (n = 1116) from the Tigre complex, a large public architectural group on the site's western edge (Fig. 2). Most of the turkey bones were associated with the Jaguar Paw Temple (Op. 26), a nine meter high platform topped by triadic architecture and decorated with sculptured stucco masks. An additional turkey specimen was recovered from an eight meter high building (Op. 35) located on the east side of the Tigre Plaza. The turkey bones were associated with Late Preclassic ceramics in well-sealed, undisturbed contexts [14] (Text S1, Fig. S2, Fig. S3, Fig. S4). AMS radiocarbon ages from animal bones found in close association with the turkey remains confirm that the deposits are Preclassic (cal 327 BC–AD 54) (Table S2).

## Results

**Figure 3:**
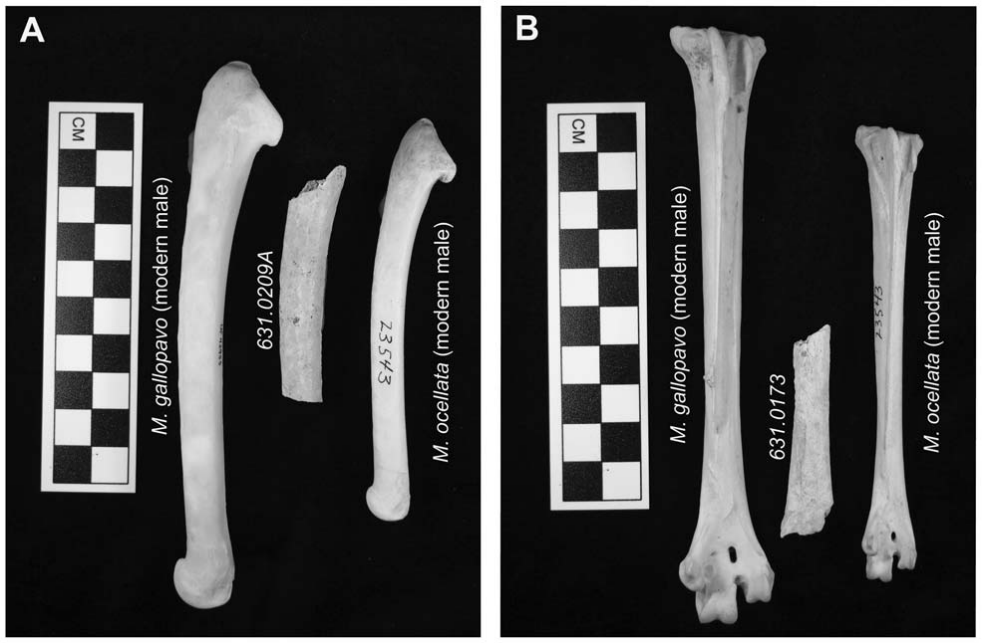
Archaeological turkey specimens compared with modern *M. gallopavo* and *M. ocellata*: A) right ulnae, and B) left tarsometatarsi.

The turkey specimens were identified during zooarchaeological analysis of El Mirador animal remains conducted by the Environmental Archaeology Program of the Florida Museum of Natural History (FLMNH-EAP), University of Florida. Comparison with modern FLMNH-EAP and FLMNH-Ornithology collections confirmed that six of the seven specimens are *M. gallopavo* (Table 1). The remaining specimen (a fragmentary femur) could not be identified to the species level because of poor preservation. Morphological characteristics identifying the specimens as *M. gallopavo* include element size, shape/curvature and robustness as well as, on two ulnae, spacing of the quill tubercles (also called cubital tubercles or papillae remigiales) (Fig. 3). The quill tubercles, which form where tendons connect the secondary flight feathers to the ulna, are also underdeveloped, suggesting reduced flight activity and thus captive rearing. Age and sex characteristics (e.g., skeletal element size, tarsometatarsus spur morphology) indicate that a minimum of three Mexican turkeys are represented in the assemblage—two males and a female. One of the males is a subadult (<2 years old). The presence of male, female, adult and subadult individuals further supports the suggestion of captive rearing.

**Table 1:** Provenience dates and zooarchaeological, aDNA and osteometric identifications of the El Mirador turkey specimens.

Catalog no.	Provenience	AMS date (calibrated)^a^	Zooarchaeological identification	aDNA identification	Osteometric identification	Element	Sex/Age
631.0209A	26O-25/27	200 BC–AD 3	*M. gallopavo*	*M. gallopavo*	*M. gallopavo*	Ulna	male (adult)
631.0173	26J-4	-	*M. gallopavo*	*M. gallopavo*	*M. gallopavo*	tarsometatarsus	male (subadult)
631.0152	26J-14	186 BC–AD 54	*M. gallopavo*	no amplification	*M. gallopavo*	ulna	male (adult)
631.0206	35B-5	327–204 BC	*M.* cf. *gallopavo*	*M. gallopavo* b	inconclusive	carpometacarpus	-
631.0210	26O-25/27	200 BC–AD 3	*M.* cf. *gallopavo*	not tested	inconclusive	carpometacarpus	-
631.0209B	26O-25/27	200 BC–AD 3	*M. gallopavo*	not tested	-	tarsometatarsus	female
631.0341	26K-4	-	*Meleagris*. sp.	not tested	-	femur	-

a AMS dates from zooarchaeological specimens found in association with the turkey bones (Table S2).

b aDNA identification was confirmed through repeat extractions and amplification.

**Figure 4:**
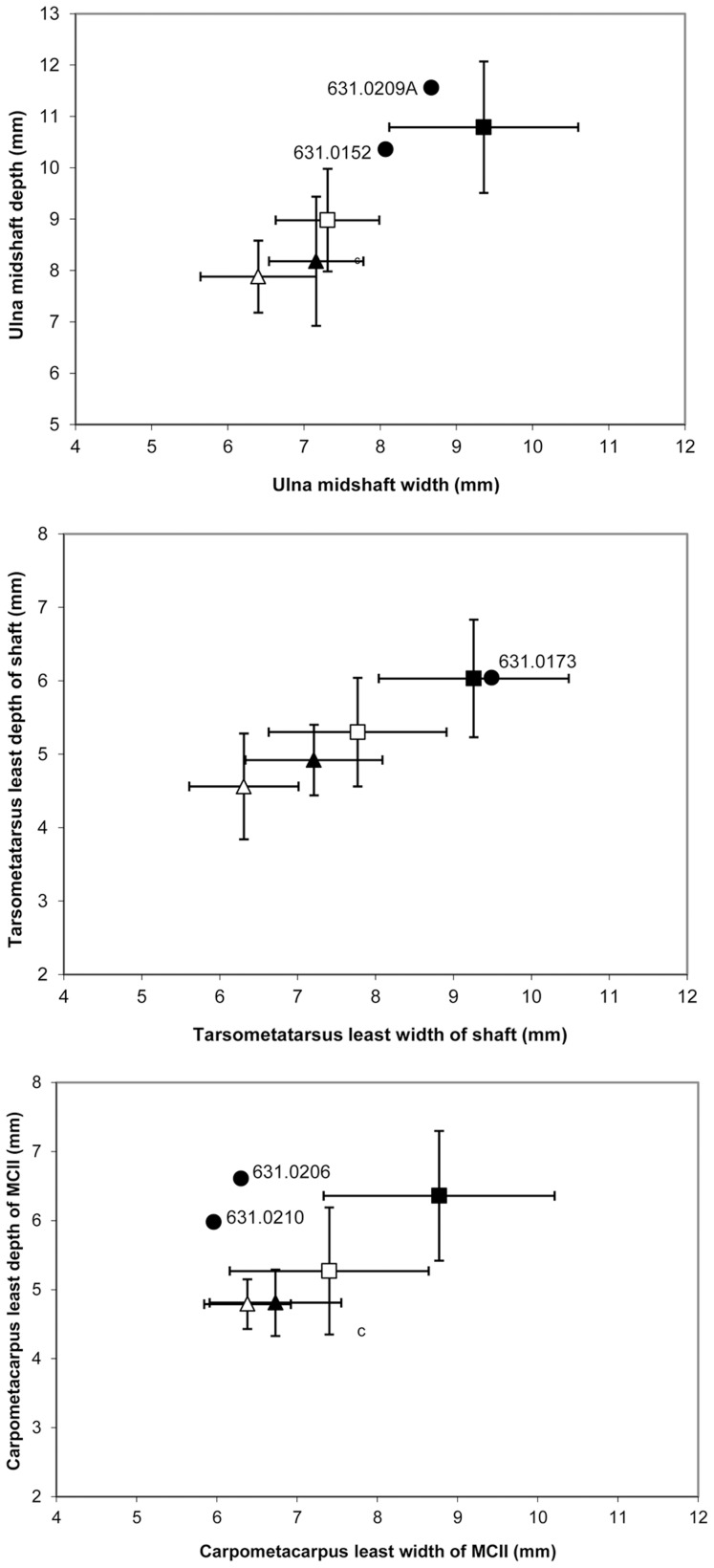
Archaeological turkey (•) osteometrics compared with mean (±2 standard deviations) *M. gallopavo* (male ▪, female ▴); and *M. ocellata* (male □, female ▵) [**6**].

The morphological evaluations of species, age and sex were supported by osteometric analysis. Five of the seven skeletal elements were complete enough to allow for shaft width and depth measurements. When compared to published *M. gallopavo* and *M. ocellata* osteometrics [6], three specimens fall within the range of adult male domestic Mexican turkeys (Fig. 4).

Ancient DNA analysis of four of the turkey bones, conducted in the Simon Fraser Ancient DNA Laboratory, further verified the morphological and osteometric identifications (Table 1). Poor preservation of tropical faunal assemblages is often problematic for aDNA analysis. Nonetheless, preservation was sufficient to allow numerous successful PCR amplifications of short fragments (93–120 bp) of *Meleagris* DNA using a combination of different primer sets (Table S3). A total of 80 PCR amplifications were conducted on the ancient bone samples, eight of which yielded PCR amplifications and sequences of expected length (Table S4). While three of the bones produced at least one short *Meleagris* DNA sequence, only one bone yielded replicable DNA sequences using multiple primer sets. Two different fragments of control region mitochondrial DNA (mtDNA) (121 bp and 106 bp respectively) were successfully amplified and replicated from both the initial and repeat extraction. All obtained mtDNA sequences matched most closely or identically with *M. gallopavo* sequences, and were considerably different from those of *M. ocellata* (Table S5), confirming the species identity as *M. gallopavo*.

## Discussion

### Mesoamerican Preclassic Trade Connections

The combined morphological, osteometric and aDNA evidence confirm the early presence of *M. gallopavo* at El Mirador in the Maya lowlands. The presence of male, female and subadult turkeys, some with reduced flight capabilities, suggests that the introduced birds were captive reared and/or domesticated. Although we do not yet know the immediate source of the turkey bones, the most reasonable explanation is that a few Mexican turkeys entered the site as exchange goods directly from central/northern Mexico suggesting that Late Preclassic association between El Mirador and contemporary northern Mesoamerican cultures at sites such as Teotihuacan was closer than previously recognized. Most of the evidence for the exchange of goods and ideas between central Mexico and the Maya region dates to the Classic period several centuries later (*ca.* AD 250–900) [15]. The El Mirador turkeys therefore add to a relatively sparse record of Preclassic cultural and material exchange between the Maya lowlands and northern Mesoamerica [16]–[18]. Prior information on Preclassic exchange comes primarily from non-perishable goods such as obsidian and ceramics so the non-local turkeys at El Mirador also expand our understanding of the types of goods that were exchanged long distances during this early period of Maya history. Although The El Mirador turkey specimens could represent the transport of dried meat or partial carcasses, the presence of associated upper and lower limb bones suggests that the animals were imported whole and possibly live. The imported turkeys further emphasize that El Mirador's vast Late Preclassic trade connections extended some 1000 kilometers north into central Mexico, in addition to the site's better known connections with the Atlantic and Pacific coasts and the Maya highlands [14], [19], [20]. El Mirador's participation in interregional trade and cultural interaction was likely pivotal to the site's accumulation of political and economic power during the Late Preclassic [20], [21].

### Timing of Mesoamerican Turkey Domestication and Diffusion

The Late Preclassic presence of *M. gallopavo* in the Maya region has important implications for documenting the timing of Mesoamerican turkey domestication and diffusion.

To date, morphological and genetic studies have been unable to distinguish between wild and early domestic forms of *M. gallopavo*. In the absence of morphological and genetic markers, archaeologists have relied on indirect evidence of domestication such as the presence of pen structures, egg shells and neonates or appearance of the species outside its presumed natural geographic range. Previous to our study, all indirect evidence for Mesoamerican turkey husbandry dated to the Classic period or later [8], [22], [23]. Since the Preclassic El Mirador turkeys represent movement of *M. gallopavo* outside its natural geographic range, the specimens represent the earliest indirect evidence of captive turkey rearing or domestication in Mesoamerica. A Preclassic origin for Mexican turkey domestication has been suggested previously [11], [24], but archaeological evidence has been lacking. The El Mirador turkey specimens confirm that turkey domestication, or at least captive rearing, dates to the Preclassic.

Determining when Mesoamerican cultures started experimenting with turkey rearing and domestication is vital to the larger question of whether the origins of New World turkey husbandry should be attributed to cultures of the American Southwest or Mesoamerica. It was originally believed that the turkey was first domesticated in Mesoamerica and then introduced in domestic form to the American Southwest [1]. More recent archaeological and genetic evidence has overturned this scenario demonstrating that turkeys were independently domesticated in these two regions although the timing of domestication remains unclear [9], [25], [26]. It is possible that the idea for turkey rearing or husbandry prior to domestication also arose independently in the American Southwest and Mesoamerica. However, the wealth of documented cultural and material exchange between the regions supports a model of diffusion for the concept of turkey husbandry as part of early exchange networks. The Late Preclassic El Mirador *M. gallopavo* specimens provide evidence for the antiquity of Mesoamerican turkey rearing, and support the probable role of interregional turkey exchange in the diffusion of ideas about animal management in the New World.

The presence of Late Preclassic Mexican turkeys at El Mirador also confirms that this non-local species was introduced to the Maya region over one thousand years earlier than previously thought. If the El Mirador turkeys are isolated examples of imported captive-raised/domestic Mexican turkeys, it raises the question of why the Maya did not broadly adopt the idea of turkey rearing or domestication until the Postclassic. One possibility is that turkey domestication was not widespread or common in any part of Mesoamerica until the later half of the Classic period despite its potentially earlier origins. This suggestion is supported by the relative rarity of *M. gallopavo* specimens in Preclassic/Formative central Mexican faunal assemblages, and their increasing frequency in Classic and Postclassic deposits [11], [22]. An alternative explanation is related to the nature of the Postclassic Maya economy. During the Postclassic, long-distance trade between central Mexico and the Maya area increased with the expansion of maritime trade routes around the Yucatan Peninsula between the Gulf of Mexico and Central America's Caribbean coast [27], [28]. Increased Postclassic exchange throughout Mesoamerica could have facilitated the dispersal of domesticated turkeys to the Maya area through repeated introductions of breeding pairs and transmission of rearing information. In contrast, the rare earlier introduction of the bird might not have been sufficient to fully incorporate the species into the Maya economy.

Although the El Mirador turkeys may represent isolated imports, it is also possible that *M. gallopavo* has been under-identified in Preclassic and Classic Maya zooarchaeological assemblages since *M. gallopavo* and *M. ocellata* can be difficult to differentiate morphologically when preservation is poor. Researchers also may not have considered the possible presence of *M. gallopavo* in earlier assemblages due to the longstanding belief that they were not introduced to the Maya region until the Postclassic. It is essential to determine whether the Mexican turkey appeared in the Maya region earlier than previously understood because an earlier introduction would have provided a second domestic vertebrate during the Late Preclassic to Classic period of Maya population expansion and increasing social complexity. During the Preclassic, the Maya relied extensively on the domestic dog (*Canis lupus familiaris*), which they used for both dietary and ritual purposes [29], although perhaps primarily for ceremonies related to elite display and power negotiations [29], [30]. The turkey was another important food and ritual animal among the Maya [31]. Prior models suggest that only local, wild ocellated turkeys were used through the Classic period, but an early demand for domesticated or captive-reared turkey (i.e., *M. gallopavo*) could have been related to increased elite ceremonial and status-displaying activities as well as the need for meat to feed growing populations during the Late Preclassic to Classic period of population growth and cultural florescence.

### Conclusions

Combined zooarchaeological and aDNA analyses identified the earliest non-local Mexican turkey remains in the Maya cultural region at the site of El Mirador. Prior to this discovery, the earliest evidence of *M. gallopavo* in the Maya area dated to approximately one thousand years later. The El Mirador turkeys may represent rare or isolated imports from central/northern Mexico, but it is also possible that captive/domestic Mexican turkey husbandry was practiced by the ancient Maya much earlier than previously thought. The Maya may therefore have had access to another domestic vertebrate, besides the dog, during the Late Preclassic to Classic period of population expansion and increasing social complexity. Significantly, the El Mirador turkeys also provide the earliest indirect evidence of *M. gallopavo* captive rearing or domestication in Mesoamerica. Previously, all other indirect evidence of husbandry (e.g., pen structures, egg shells and neonates, or the appearance of the species outside its natural geographic range) dated to the Classic period or later [8], [22], [23].

The early presence of *M. gallopavo* at Late Preclassic El Mirador demonstrates a need to reassess the timing of turkey domestication and diffusion in Mesoamerica. Understanding when the Mexican turkey was domesticated and when it was introduced to and fully adopted by the ancient Maya has important consequences for understanding Mesoamerica subsistence systems and long-distance trade connections. The topic also has broader ramifications with respect to the process and timing of New World animal domestication, and the culture-specific motivations for incorporating or not incorporating potential domesticates or managed species into ancient social and economic systems.

## Materials and Methods

### Zooarchaeology and Osteometrics

The archaeological turkey bones were identified within a larger zooarchaeological assemblage from the site (number of identified specimens = 3470). The sample also contained other bird bones that we could only identify to the level of taxonomic subclass (Aves) because they were undiagnostic elements or poorly preserved. Nearly all of the unidentified bird remains come from large-bodied species, and some of these may represent additional *M. gallopavo* elements.

Zooarchaeological specimens were identified through comparison with modern skeletons housed in the Florida Museum of Natural History Environmental Archaeology and Ornithology collections (www.flmnh.ufl.edu/museum/collections.htm). Turkey age and sex determinations were based on skeletal element size, osteometrics [6], [32], and tarsometatarsus spur morphology. Archaeological bones were measured using standard osteometric measurements and were compared to published metric data available for *M. gallopavo* and *M. ocellata* [6: Tables 10,11,14,15,20,21].

### Ancient DNA Analysis

The four archaeological bird bones were processed in the Ancient DNA Laboratory located in the Department of Archaeology at Simon Fraser University. The ancient DNA laboratory is specifically designed for and dedicated to ancient DNA work - no modern DNA samples have ever been processed in the lab. The lab is equipped with a UV filtered ventilation and positive airflow, with dedicated equipment and bench UV lights. Strict contamination control protocols are followed in the lab, including: 1) the use of protective clothing including Tyvex™ suits, gloves, masks, etc.; 2) the separation of the pre- and post-PCR work (located in two buildings with separate ventilation systems); and 3) the inclusion of multiple blank DNA extractions and negative PCR controls.

Two separate DNA extractions were conducted for each bone, with the repeat extractions occurring several months after the initial extractions. For both extractions, the analyzed bone samples weighed approximately 0.5 g. Bone samples were subjected to rigorous chemical decontamination in order to remove possible surface contamination [33]. The samples were immersed in a 6% sodium hypochlorite solution for 7 minutes, followed by immersion in 1 N HCl solution for 30–60 seconds, then immersion in 1 N NaOH for 30–60 seconds, before being rinsed twice in ultra-pure water and UV irradiated in a crosslinker for 30 minutes on two sides. The samples were crushed into powder using a liquid nitrogen grinding mill (6750 SPEX CertiPrep Freezer/Mill). Three additional ancient turkey bones were included in analysis to act as positive controls for both the initial and repeat extractions, as well as the subsequent PCR reaction sets. These three bones were recovered from archaeological sites in Arizona (*ca.* AD 1100–1300) [9] and were processed separately from the El Mirador samples. DNA extraction was performed using a modified silica-spin column technique [9], [34], and approximately 100 µl of DNA solution was collected for each sample.

PCR amplifications were conducted in a Mastercycler Personal (Eppendorf, Hamburg, Germany) in a 30–50 µL reaction volume containing 50 mM KCl, 10 mM Tris-HCl, 2.5 mM MgCl2, 0.2 mM dNTP, 1.0 mg/ml BSA, 0.3 µM each primer, 3.0–5.0 µl DNA sample and 2.5–3.5 U AmpliTaq Gold™ LD (Applied Biosystems). Primers were designed to target fragments of *Meleagris* mitochondrial DNA of various lengths. Several different primer sets were tested (Table S3). PCR began with an initial 12 minute denaturing period at 95°C, followed by 60 cycles at 94°C for 30 seconds (denaturing), 52°C for 30 seconds (annealing), and 72°C extension for 40 seconds. Blank extracts and negative controls were included in each of the PCR reaction sets. Ancient positive controls (Arizona archaeological turkey bone extracts) were also tested to ensure the efficacy of the primer sets and PCR conditions.

Five µL of PCR product were visualized via electrophoresis on a 2% agarose gel using SYBR Green™ staining. Successfully amplified PCR products of expected length were purified using MinElute™ purification kits (Qiagen, Valencia, CA). Purified products were sequenced using both forward and reverse primers at the Central Facility of the Institute for Molecular Biology and Biotechnology Laboratory at McMaster University (using an ABI 3100) and at Macrogen, Seoul, Korea (ABI 3730XL). The obtained electropherograms were edited, aligned and compiled using ChromasPro software (www.technelysium.com.au). Consensus sequences were developed based on multiple PCR amplifications and sequencing.

Once the DNA analysis of the ancient samples was completed, DNA was extracted from a modern *M. ocellata* phalanx collected from Guatemala (FLMNH catalog number Z11050, Table S4). The *M. ocellata* samples were processed in the SFU Center for Forensic Research in a lab dedicated to DNA analysis of modern or forensic bone samples. Two 0.5 g bone samples were extracted using methods similar to those listed above. The DNA extracts were PCR amplified using primers TK-F2/TK-R405 and TK-F252/TK-R567 (Table S3) and produced amplicons of 254 bp and 338 bp in length, respectively. The PCR products were sequenced from both directions and consensus sequences matched identically with the GenBank *M. ocellata* reference sequence AF487120.

### Ancient DNA Extraction Results and Authenticity

Sequences were obtained for three (631.0209, 631.0173, 631.0206) of the four bone samples. Only one bone yielded replicable sequences (631.0206) using different primer sets and using both the initial and repeated DNA extracts (Table S4). The obtained ancient DNA sequences were BLAST-compared through GenBank to determine if they would match *Meleagris* sequences and to ensure that they did not match with any other unexpected species or sequences. Multiple alignments of the sample sequences and published *Meleagris* mtDNA reference sequences were conducted using ClustalW [35] in order to confirm the species identifications.

Two replicable DNA fragments were obtained for sample 631.0206 totaling 71 bp and 55 bp respectively once the primer sequences were removed. The two fragments correspond to positions 15731–15800, and 15858–15913 on the *M. gallopavo* mitochondrial genome (reference NC010195). Despite their short lengths, the sequences clearly match most closely with *M. gallopavo* rather than *M. ocellata*, as demonstrated by the multiple alignment (Table S5).

Due to the antiquity of the samples and the tropical climate from which they were recovered, a low success rate for DNA extraction and amplification is expected, and provides support for the authenticity of the recovered sequences. The successfully reproduced sample, the carpometacarpus fragment, was the largest of the four bone samples and the best preserved morphologically [36]. PCR targeted fragments of mtDNA varying in length from <100 to 400 bp. Only short fragments of DNA could be amplified despite repeat amplifications with longer primer sets designed to detect contamination from modern sources [37]. Multiple blank extractions and negative PCR controls were included in the study, none of which yielded DNA fragments of expected length. Successful amplification of three positive controls (ancient turkey bones from Arizona) demonstrated the efficacy of both the extraction method and PCR primers [9].

The retrieved sequences matched very closely or identically with modern turkey reference sequences in GenBank, and therefore cannot be used to definitively rule out the possibility of contamination from modern sources. However, considering the short length of the retrieved sequences significant differences between the ancient and modern turkeys were not expected. Moreover, the primer sets were designed to target areas maximizing differences between *M. gallopavo* and *M. ocellata*, rather than polymorphic sites within the *M. gallopavo* control region. The retrieved sequences themselves, including those unreplicated sequences, demonstrate significant *post-mortem* damage characterized by C→T transitions (Fig. S1). These DNA transitions were likely caused by hydrolytic damage and are anticipated to occur in ancient sequences [37], [38]. Finally, the DNA identification of *M. gallopavo* supports the morphological and osteometric identification of the bones.

## Supporting Information

Text S1 **El Mirador excavation history, provenience descriptions and dating.**
(DOCX)

Figure S1 **Multiple alignments of obtained sequences demonstrating DNA damage induced transitions.**
(DOCX)

Figure S2 **Operation 26J baulk profile. Redrawn after** [**14: Fig. 29**] **.**
(DOCX)

Figure S3 **Operation 26O baulk and tunnel profile. Redrawn after** [**14: Fig. 44**] **.**
(DOCX)

Figure S4 **Operations 35A and 35B wall and baulk profile.** Redrawn after [14: Fig. 56].
(DOCX)

Table S1 **Generalized chronology used in the text.**
(DOCX)

Table S2 **AMS Radiocarbon ages from zooarchaeological remains found in association with the archaeological turkey bones.**
(DOCX)

Table S3 ***Meleagris*** **primers for PCR amplification.**
(DOCX)

Table S4 Results of PCR amplification and sequence analysis.
(DOCX)

Table S5 **Multiple alignment of** ***M. gallopavo*** **and** ***M. ocellata*** **control-region reference sequences, with the retrieved ancient sequence.**
(DOCX)

## References

[1] Leopold AS (1959) Wildlife of Mexico: the game birds and mammals. Berkeley: University of California Press. 581.

[2] Schorger AW (1966) The wild turkey: its history and domestication. Norman: University of Oklahoma Press. 625.

[3] Howell SNG, Webb S (1995) A guide to the birds of Mexico and northern Central America. New York: Oxford University Press. 1010.

[4] Camacho-Escobar MA, Jiménez-Hidalgo E, Arroyo-Ledezma J, Sánchez-Bernal EI, Pérez-Lara E (2011) Natural history, domestication and distribution of the turkey (*Meleagris gallopavo*) in Mexico. Revista Universidad y Ciencia 27: 351–360.

[5] Corona-M E (2002) The Pleistocene bird record of México. Acta Zoologica Cracoviensia 45 special issue 293–306.

[6] Steadman DW (1980) A review of the osteology and paleontology of turkeys (Aves: Meleagridae). Contribution of the Science and Natural History Museum of Los Angeles County California 330: 131–207.

[7] Steadman DW, Stull JA, Eaton SW (1979) Natural history of the ocellated turkey. World Pheasant Assoc J 4: 15–37.

[8] Hamblin NL (1984) Animal use by the Cozumel Maya. Tuscon: University of Arizona Press. 206.

[9] Speller CF, Kemp BM, Wyatt SD, Monroe C, Lipe WD, et al (2010) Ancient mitochondrial DNA analysis reveals complexity of indigenous North American turkey domestication. Proc Natl Acad Sci USA 107: 2807–2812. 20133614 10.1073/pnas.0909724107 PMC2840336

[10] Álvarez T, Ocaña A (1999) Sinopsis de restos arqueo-zoológicos de vertebrados terrestres - basada en informes del laboratorio de paleozoología del INAH. Mexico City: INAH Colección Científica. 108.

[11] Flannery KV (1967) Vertebrate fauna and hunting patterns. In Byers DS, editor. The prehistory of the Tehuacan Valley, volume 1.Austin: University of Texas Press. 132–177.

[12] Middleton WD, Feinman GM, Nicholas LM (2002) Domestic faunal assemblages from the Classic period Valley of Oaxaca, Mexico: a perspective on the subsistence and craft economies. J Archaeol Sci 29: 233–249.

[13] Götz CM (2008) Coastal and inland patterns of faunal exploitation in the prehispanic northern Maya lowlands. Quat Int 191: 154–169.

[14] Hansen RD (1990) Excavations in the Tigre Complex El Mirador, Petén, Guatemala. Provo, UT: New World Archaeological Foundation. 308.

[15] Braswell GE, editor. The Maya and Teotihuacan: reinterpreting Early Classic interaction. Austin: University of Texas Press. 441.

[16] Moholy-Nagy H, Nelson FW (1990) New data on sources of obsidian from Tikal, Guatemala. Ancient Mesoam 1: 71–80.

[17] Pendergast DM (1971) Evidence of early Teotihucan-lowland Maya contact at Altun Ha. Am Antiq 36: 455–460.

[18] Clayton SC (2005) Interregional relationships in Mesoamerica: interpreting Maya ceramics at Teotihuacan. Lat Am Antiq 16: 427–448.

[19] Neff H, Bishop RL, Arnold DE (1990) A reexamination of the compositional affiliations of Formative period whiteware from highland Guatemala. Ancient Mesoam 1: 171–180.

[20] Matheny RT, Matheny DG (2011) Introduction to Investigations at El Mirador, Peten, Guatemala. El Mirador Series, Part 1. Provo, UT: New World Archaeological Foundation, Brigham Young University. 240.

[21] Reese-Taylor K, Walker DS (2002) The passage of the Late Preclassic into the Early Classic. In Masson M, Freidel DA, editors. Ancient Maya political economies.New York: AltaMira Press. 87–122.

[22] Lapham HA, Feinman GM, Nicholas LM (in press) Animal economies in prehispanic southern Mexico. In Götz C, Emery KF, editors. Archaeology of Ancient Mesoamerican Animals.Oxford: Oxbow Press, David Brown Book Company.

[23] Storey R (1992) Life and death in the ancient city of Teotihuacan: a modern paleodemographic synthesis. Tuscaloosa: University of Alabama Press. 328.

[24] Valadez AR (2003) Domesticación y zootecnia en el México antiguo. Imagen Veterinaria 3: 32–45.

[25] Breitburg E (1993) The evolution of turkey domestication in the greater Southwest and Mesoamerica. In Woosley AI, Revesloot JC, editors. Culture and contact: Charles C. Di Peso's gran chichimeca.Albuquerque: University of New Mexico Press. 153–172.

[26] McKusick CR (2001) Southwest birds of sacrifice. Tucson: Arizona Archaeological Society. 208.

[27] Guderjan TH, Garber JF (1995) Maya maritime trade, settlement, and populations on Ambergris Caye, Belize. San Antonio, TX: Maya Research Program and Labyrinthos. 201.

[28] McKillop H, Healy PF (1989) editors (1989) Coastal Maya trade. Peterborough, ON: Trent University Occasional Papers in Anthropology, No. 8. 189.

[29] Emery KF (2004) In search of the “Maya” diet: is regional comparison possible in the Maya area? Archaeofauna 13: 37–56.

[30] Teeter WG (2004) Animal utilization in a growing city, vertebrate exploitation at Caracol, Belize. In Emery KF, editor. Maya zooarchaeology: new directions in method and theory.Los Angeles: Institute of Archaeology, UCLA Press. 177–191.

[31] Pohl MD, Feldman LH (1982) The traditional role of women and animals in lowland Maya economy. In Flannery KV, editor. Maya subsistence: studies in memory of Dennis E. Puleston.New York: Academic Press. 295–311.

[32] von den Driesch A (1976) A guide to the measurement of animal bones from archaeological sites. Cambridge, MA: Peabody Museum of Archaeology and Ethnology, Harvard University. 137.

[33] Yang DY, Cannon A, Saunders SR (2004) DNA species identification of archaeological salmon bone from the Pacific Northwest Coast of North America. J Archaeol Sci 31: 619–631.

[34] Yang DY, Eng B, Waye JS, Dudar JC, Saunders SR (1998) Improved DNA extraction from ancient bones using silica-based spin columns. Am J Phys Anthro 105: 539–543. 10.1002/(SICI)1096-8644(199804)105:4<539::AID-AJPA10>3.0.CO;2-1 9584894

[35] Thompson JD, Higgins DG, Gibson TJ (1994) CLUSTAL W: improving the sensitivity of progressive multiples sequence alignments through sequence weighting, position-specific gap penalties and weight matrix choice. Nucl Acids Res 22: 4673–4680. 7984417 10.1093/nar/22.22.4673 PMC308517

[36] Haynes S, Searle JB, Bretman A, Dobney KM (2002) Bone preservation and ancient DNA: the application of screening methods for predicting DNA survival. J Archaeol Sci 29: 585–592.

[37] Paabo S (1989) Ancient DNA: extraction, characterization, molecular-cloning, and enzymatic amplification. Proc Natl Acad Sci USA 86: 1939–1943. 2928314 10.1073/pnas.86.6.1939 PMC286820

[38] Gilbert MT, Binladen J, Miller W, Wiuf C, Willersley E, et al (2007) Recharacterization of ancient DNA miscoding lesions: insights in the era of sequencing-by-synthesis. Nucl Acids Res 35: 1–10. 16920744 10.1093/nar/gkl483 PMC1802572

