# Supplementary material for: Earliest Mexican Turkeys (Meleagris gallopavo) in the Maya Region: Implications for Pre-Hispanic Animal Trade and the Timing of Turkey Domestication
Source: PLoS One. 2012 Aug 8;7(8):e42630. doi: 10.1371/journal.pone.0042630 (PMC3414452; doi:10.1371/journal.pone.0042630)
Supplement: Text S1 — El Mirador excavation history, provenience descriptions and dating. (DOCX) [file pone.0042630.s001.docx]

**Supporting Information (SI) Text**

***El Mirador: excavation history, provenience descriptions, and dating***

El Mirador, an ancient Maya city located deep in the tropical forest of the northern area of the Department of Petén, Guatemala is famous for its Preclassic cultural remains (Table S1). In 1932 the 16^th^ Carnegie Institution Central American Expedition briefly visited the site. Years later many of the largest architectural features of the site were mapped by Ian Graham and some areas briefly tested by Joyce Marcus. A larger program of excavation and mapping was carried out by Bruce Dahlin and Ray Matheny from 1978–1983 and included the Harvard El Mirador Project in 1982. Excavations were resumed in 2003 by Richard Hansen and are ongoing as part of what is known as the Mirador Basin investigations. The archaeological turkey specimens reported here were recovered along with other archaeological animal remains from excavations between 1980 and 1982 (Fig. 2). These materials were housed by the Brigham Young University Museum of Peoples and Cultures prior to zooarchaeological analysis of the El Mirador animal remains at FLMNH-EAP, which began in 2003.

All of the turkey bones recovered in the 1980s came from investigations in the Late Preclassic Tigre Complex, whose major focus is the 56 m tall Tigre Pyramid. Operation 26J, excavated by Hansen [14] under supervision by Matheny, was a tunnel placed under an ancient plaster floor exposed at the base of the east side of the Jaguar Paw Temple. This floor was of interest because it appeared to run under the building platform. Hansen probed under the floor with a 3.7 m tunnel, encountering a second continuous floor (below the first) that sealed a still-lower midden layer containing bones, pieces of charcoal, ceramics, and other cultural materials. Turkey bones were found in Lots 04 and 14, and radiocarbon sample Beta 241842 was recovered from Lot 14 (Fig. S2). Hansen [14: Table 4] classified all ceramics from the lots below the lower floor as Preclassic. The midden layer may have been in place prior to floor construction or brought in from elsewhere as floor preparation fill. Below the midden layer, excavation continued through a substantial layer of sterile sascab (deteriorated limestone) lying on bedrock.

Operation 26O was an exploratory 33 m tunnel into the heart of the Jaguar Paw Temple [14: 70–73]. The turkey bones, and the mammal bone used for radiocarbon sample Beta 241844, were found in contiguous Lots 25/27. The specimens were found about 24 meters into the structure, almost directly under the center of the temple above (Fig. S3). The context of the bones was sealed in antiquity first by Middle Preclassic architectural remains and then by Late Preclassic construction of the temple.

Operations 35 A and B were in Structure 4D2-1, a prominent 8 m high building located on the east side of the Tigre Plaza, directly east of the main Tigre Pyramid stairway (Fig. 1). This building is the central construction of a triadic group. Operation 35A included the excavation of two large chambers on the summit of the building, including the central room [14, Fig. S4]. Excavations continued below what may have been the latest floor in the room. In Operation 35B, which was a continuation of Operation 35A to a deeper level in the southern portion of the central room, plaster floors were found at -3.18 m and at -4.7 m below datum. The excavations continued beneath the lower plaster floor to -9.05 m in Lot 05 where radiocarbon sample Beta 241843 and turkey bones were found. Hansen [14] reported that all of the estimated 400 potsherds from the sealed context of Operation 35B were from the early Late Preclassic period.
