# Supplementary material for: Earliest Mexican Turkeys (Meleagris gallopavo) in the Maya Region: Implications for Pre-Hispanic Animal Trade and the Timing of Turkey Domestication
Source: PLoS One. 2012 Aug 8;7(8):e42630. doi: 10.1371/journal.pone.0042630 (PMC3414452; doi:10.1371/journal.pone.0042630)

**Figure S1:** Multiple alignments of obtained sequences demonstrating DNA damage induced transitions.


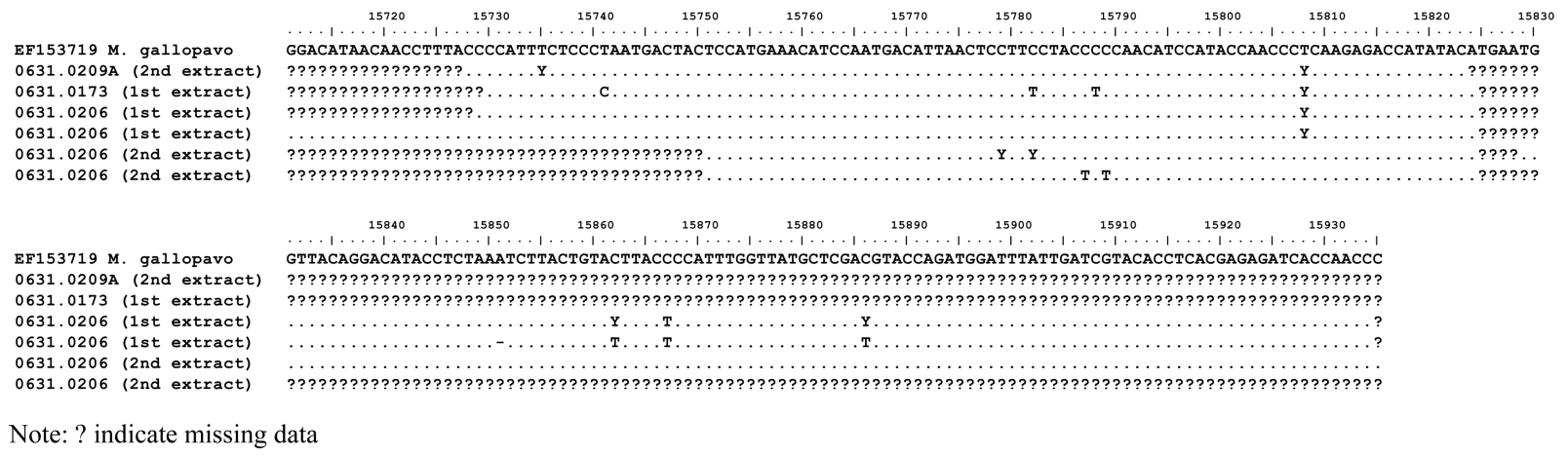

Supplement: Figure S1 — Multiple alignments of obtained sequences demonstrating DNA damage induced transitions. (DOCX) [file pone.0042630.s002.docx]
