# Supplementary material for: Earliest Mexican Turkeys (Meleagris gallopavo) in the Maya Region: Implications for Pre-Hispanic Animal Trade and the Timing of Turkey Domestication
Source: PLoS One. 2012 Aug 8;7(8):e42630. doi: 10.1371/journal.pone.0042630 (PMC3414452; doi:10.1371/journal.pone.0042630)
Supplement: Figure S4 — Operations 35A and 35B wall and baulk profile. Redrawn after [14: Fig. 56]. (DOCX) [file pone.0042630.s005.docx]

**Figure S4:** Operations 35A and 35B wall and baulk profile. Redrawn after [14: Fig. 56].
