# Supplementary material for: Earliest Mexican Turkeys (Meleagris gallopavo) in the Maya Region: Implications for Pre-Hispanic Animal Trade and the Timing of Turkey Domestication
Source: PLoS One. 2012 Aug 8;7(8):e42630. doi: 10.1371/journal.pone.0042630 (PMC3414452; doi:10.1371/journal.pone.0042630)
Supplement: Table S1 — Generalized chronology used in the text. (DOCX) [file pone.0042630.s006.docx]

**Table S1:** Generalized chronology used in the text.

| Mesoamerican cultural period | Approximate dates |
| --- | --- |
| Early Preclassic/Formative | 2000–1000 BC |
| Middle Preclassic/Formative | 1000–300 BC |
| Late Preclassic/Formative | 300 BC–AD 100 |
| Terminal Preclassic/Protoclassic | AD 100–250 |
| Early Classic | AD 250–600 |
| Late Classic | AD 600–800 |
| Terminal Classic | AD 800–1000 |
| Postclassic | AD 1000–1500 |
