# Supplementary material for: Earliest Mexican Turkeys (Meleagris gallopavo) in the Maya Region: Implications for Pre-Hispanic Animal Trade and the Timing of Turkey Domestication
Source: PLoS One. 2012 Aug 8;7(8):e42630. doi: 10.1371/journal.pone.0042630 (PMC3414452; doi:10.1371/journal.pone.0042630)
Supplement: Table S2 — AMS Radiocarbon ages from zooarchaeological remains found in association with the archaeological turkey bones. (DOCX) [file pone.0042630.s007.docx]

**Table S2:** AMS Radiocarbon ages from zooarchaeological remains found in association with the archaeological turkey bones.

| Taxa  (scientific name) | Beta Analytic  lab no. | Provenience  (Op. no.) | Uncalibrated ages (years BP) | 2 Sigma calibrated radiocarbon ages (INTCAL09 calendar years) |
| --- | --- | --- | --- | --- |
| *Odocoileus virginianus* | Beta-241842 | 26J-14 | 2050 +/- 50 | 186 BC–AD 54 |
| Mammalia | Beta-241843 | 35B-5 | 2250 +/- 40 | 330 BC–204 BC |
| Mammalia | Beta-241844 | 26O-25/27 | 2080 +/- 40 | 200 BC–AD 3 |
