# Supplementary material for: Earliest Mexican Turkeys (Meleagris gallopavo) in the Maya Region: Implications for Pre-Hispanic Animal Trade and the Timing of Turkey Domestication
Source: PLoS One. 2012 Aug 8;7(8):e42630. doi: 10.1371/journal.pone.0042630 (PMC3414452; doi:10.1371/journal.pone.0042630)
Supplement: Table S3 — Meleagris primers for PCR amplification. (DOCX) [file pone.0042630.s008.docx]

**Table S3:** *Meleagris* primers for PCR amplification.

| Primer name | Direction | Coordinates^§^ | Sequence (5' to 3') |
| --- | --- | --- | --- |
| TK-F2* | Forward | 15482–15505 | AATTTATTCCCGCTTGGATAAGCC |
| TK-F143* | Forward | 15624–15650 | GCATAATCGTGCATACATTTATATACC |
| TK-F205 | Forward | 15685-15712 | CGTACTAAACCCATTATATGTARACGGA |
| TK-F224* | Forward | 15704–15729 | GTAGACGGACATAACAACCTTTACCCC |
| TK-F247 | Forward | 15729–15754 | CCCATTYCTCCCTAATGACTACTCC |
| TK-F252 | Forward | 15731-15755 | CCATTTCTCCCACAATGACTACTCC |
| TK-F315* | Forward | 15759–15782 | ACATGCCAATGACATTAACTCCTTC |
| TK-F411* | Forward | 15829–15854 | TGGTTACAGGACATACCTCTAAATCT |
| TK-R261* | Reverse | 15718–15741 | AGGGAGRAATGGGGTAAAGGTTGT |
| TK-R405* | Reverse | 15801–15824 | TGTATATGGTCTCTTGRGGGTTGG |
| TK-R519 | Reverse | 15914–15935 | GGGTTGGTGATCTCTCGTGAGG |
| TK-R567* | Reverse | 15962–15981 | GGGAAAGAATGGGCCTGAAG |

* Previously published results [9]

^§^ Coordinates numbered according to GenBank reference sequence accession EF153719.
