# Supplementary material for: Earliest Mexican Turkeys (Meleagris gallopavo) in the Maya Region: Implications for Pre-Hispanic Animal Trade and the Timing of Turkey Domestication
Source: PLoS One. 2012 Aug 8;7(8):e42630. doi: 10.1371/journal.pone.0042630 (PMC3414452; doi:10.1371/journal.pone.0042630)
Supplement: Table S4 — Results of PCR amplification and sequence analysis. (DOCX) [file pone.0042630.s009.docx]

**Table S4:** Results of PCR amplification and sequence analysis. This table lists the ratio of successfully PCR amplifications

compared to total number of PCR attempts for all samples.

| Catalog No. | Extraction | TK-F2/ TK-R405 (342bp) | TK-F252/ TK-R567 (250bp) | TK-F143/ TK-R405 (200bp) | TK-F315/ TK-R519 (176bp) | TK-F205/ TK-R405 (139bp) | TK-F224/ TK-R405 (120bp) | TK-F143/ TK-R261 (117bp) | TK-F411/ TK-R519 (106bp) | TK-F247/ TK-R405 (95bp) | TK-F252/ TK-R405 (93bp) | TOTAL PCRs | Species Identity |
| --- | --- | --- | --- | --- | --- | --- | --- | --- | --- | --- | --- | --- | --- |
| 631.0152 | 1st | 0/1 |  | 0/1 | 0/1 | 0/1 | 0/2 | 0/1 | 0/2 | 0/2 | 0/2 | 0/13 | N/A |
|  | 2nd |  |  |  | 0/1 | 0/1 | 0/1 | 0/1 | 0/1 | 0/1 | 0/1 | 0/7 |  |
| 631.0209A | 1st | 0/1 |  | 0/1 | 0/1 | 0/1 | 0/2 | 0/1 | 0/2 | 0/2 | 0/2 | 0/13 | *M. gallopavo* |
|  | 2nd |  |  |  | 0/1 | 0/1 | 0/1 | 0/1 | 0/1 | **1/1** | 0/1 | **1/7** |  |
| 631.0173 | 1st | 0/1 |  | 0/1 | 0/1 | 0/1 | 0/2 | 0/1 | 0/2 | **1/2** | **0/2** | **1/13** | *M. gallopavo* |
|  | 2nd |  |  |  | 0/1 | 0/1 | 0/1 | 0/1 | 0/1 | 0/1 | 0/1 | 0/7 |  |
| 631.0206 | 1st | 0/1 |  | 0/1 | 0/1 | 0/1 | **1/2** | 0/1 | **1/2** | **1/2** | **1/2** | **4/13** | *M. gallopavo* |
|  | 2nd |  |  |  | 0/1 | 0/1 | 0/1 | 0/1 | **1/1** | 0/1 | **1/1** | **2/7** |  |
| Z11050 | 1st | 2/2 | 2/2 |  |  |  |  |  |  |  |  | **4/4** | *M. ocellata* |
|  | 2nd | 1/2 | 1/2 |  |  |  |  |  |  |  |  | **2/4** |  |

Note: Specimen Z11050 is from a modern *M. ocellata* collected in the Guatemalan Petén region and is curated as part of the modern comparative

collections of the FLMNH-EAP. All other specimens are archaeological remains from the site of El Mirador (FLMNH-EAP accession number 631).
