# Supplementary material for: Earliest Mexican Turkeys (Meleagris gallopavo) in the Maya Region: Implications for Pre-Hispanic Animal Trade and the Timing of Turkey Domestication
Source: PLoS One. 2012 Aug 8;7(8):e42630. doi: 10.1371/journal.pone.0042630 (PMC3414452; doi:10.1371/journal.pone.0042630)
Supplement: Table S5 — Multiple alignment of M. gallopavo and M. ocellata control-region reference sequences, with the retrieved ancient sequence. (DOCX) [file pone.0042630.s010.docx]

**Table S5:** Multiple alignment of *M. gallopavo* and *M. ocellata* control-region reference sequences, with the retrieved ancient sequence.

|  | 15740 | 15741 | 15741.1 | 15760 | 15761 | 15762 | 15762.1 | 15763 | 15776 | 15777 | 15778 | 15788 | 15789 | 15790 | 15791 | 15794 | 15795 | 15796 | 15799 | 15860 | 15863 | 15885 | 15896 |
| --- | --- | --- | --- | --- | --- | --- | --- | --- | --- | --- | --- | --- | --- | --- | --- | --- | --- | --- | --- | --- | --- | --- | --- |
| NC010195 M. *gallopavo* | C | T | - | C | A | T | - | C | C | T | C | - | C | C | C | A | T | C | T | T | C | A | G |
| AF532414 *M. gallopavo* | A | - | - | . | . | . | G | . | . | . | . | - | . | . | . | . | . | . | . | . | . | . | . |
| AJ297180 *M. gallopavo* | . | . | - | . | . | . | - | . | . | . | . | - | . | . | . | . | . | . | . | . | . | . | A |
| AF486875 *M.g.silvestris* | . | . | - | . | . | . | - | . | . | . | . | - | . | . | . | . | . | . | . | . | . | . | . |
| GQ303165 *M.g.gallopavo* | . | . | - | . | . | . | - | . | . | . | . | - | . | . | . | . | . | . | . | . | . | . | . |
| AF487121 *M.ocellata* | . | A | C | T | G | C | - | T | T | C | T | C | A | T | A | C | C | T | G | C | G | - | . |
| AF487120 *M.ocellata* | . | A | C | T | G | C | - | T | T | C | T | C | A | T | A | C | C | T | G | C | G | - | . |
| *M. ocellata* (cat #Z11050) | . | A | C | T | G | C | - | T | T | C | T | C | A | T | A | C | C | T | G | C | G | - | . |
| Specimen 631.0206 | . | . | - | . | . | . | - | . | . | . | . | - | . | . | . | . | . | . | . | . | . | . | . |
